# Cultivating the Interpersonal Domain: Compassion in the Supervisor-Doctoral Student Relationship

**DOI:** 10.3389/fpsyg.2021.567664

**Published:** 2021-04-30

**Authors:** Oskar Lundgren, Walter Osika

**Affiliations:** ^1^Division of Children’s and Women’s Health, Crown Princess Victoria Children’s Hospital, Linköping, Sweden; ^2^Department of Biomedical and Clinical Sciences, Faculty of Medicine and Health Sciences, Linköping University, Linköping, Sweden; ^3^Department of Neurobiology, Care Sciences and Society, Center for Social Sustainability, Karolinska Institute, Stockholm, Sweden; ^4^Department of Clinical Neuroscience, Center for Psychiatry Research, Karolinska Institute & Stockholm Health Care Services, Stockholm, Sweden

**Keywords:** doctoral student supervision, compassion, interpersonal relationship, conceptual paper, post graduate student

## Abstract

The long-term and complex supervisor-doctoral student relationship is often characterised by tension and frictions. In higher education research, models, and interventions that take the potential beneficial interpersonal effects of compassion into account seem to be scarce. Hence, the aim of this study was to conceptualise the potential role compassion could have in the cultivation of an affiliative and sustainable supervisor-doctoral student relationship. The concept of compassion was investigated and analysed in relation to a contemporary model of supervisor behaviours. Furthermore, a systematic literature search in the scientific databases PubMed, PsychInfo, ScienceDirect, and Google Scholar was performed. The conceptual analysis revealed that the interpersonal domain, in which compassion could afford a shared sense of warmth, is neglected in previous definitions. Furthermore, the integration of compassion into a model of adaptive supervisor behaviour indicates a strong case for a salutary role for compassion in the supervisor-doctoral student relationship. However, the literature review showed that empirical data are lacking, and more studies are needed. The role of compassion deserves to be investigated empirically in this particular interpersonal context.

## Introduction

### The Supervisor-Doctoral Student Relationship

From the outside, doctoral studies might look like a dream come true for the lucky students who walk the academic path to a doctorate degree. What could be the downside of getting paid for reading, experimenting, thinking, and writing about a favourite subject, and then getting rewarded with a PhD title in the end? Well, it is actually possible to imagine that the long-term and complex relationship between students and their supervisors could turn into a source of friction. Indeed, research within the field of higher education show that relational pitfalls, disagreements, conflict, and other ethical issues are some of the main hindrances for progress with the academic work, and sometimes even contribute to mental health issues (Bergnéhr, 2013; Gunnarson et al., 2013; Moxham et al., 2013; Holbrook et al., 2014; Löfström and Pyhältö, 2014; Levecque et al., 2017; Corcelles et al., 2019). On the other hand, supervision experienced as supportive is associated with less emotional exhaustion among students (Devine and Hunter, 2017). From the supervisor’s point of view, interpersonal difficulties are commonly experienced, although this facet of the relationship seems to be less well studied (Lindén, 1999).

As such difficulties are rather common, a variety of courses and development programmes for supervisors as preventive measures have been provided, and some also investigated (Lindén, 1999; Pearson and Brew, 2002). Among these, only a few have included the relational dimension of the supervisor role, and courses that use the latest tools and findings from clinical and social psychology seem to be rare (Lindén, 1999). One such example is a program developed by Manathunga (2005) called “Compassionate Rigour”. However, the programme uses “compassion” as an umbrella term for being supportive in general and does not contain more specific aspects of compassion (Manathunga, 2005). Hence, findings from the recently sprouting literature on the influence of compassion on health and well-being (Gilbert, 2017; Seppelä et al., 2017; Kirby, 2017), and its potential to cultivate trust and cooperation in the workplace (Lilius et al., 2008), as well as to improve the classroom milieu in schools (Jazaieri, 2018) is not yet integrated into higher education curricula. This might reflect a substantial gap in knowledge.

### The Concept of Compassion

The concept of compassion has played an essential role in diverse traditions such as Greek philosophy, Buddhist psychology, ethics, and contemporary moral philosophy (Fröding and Osika, 2015; Gilbert, 2017; Seppelä et al., 2017; White, 2017). Furthermore, the roots of compassion and its precursor empathy have been investigated and debated from evolutionary and biological perspectives (Gilbert, 2005; Keltner, 2009; De Waal, 2010; Goetz et al., 2010; Zaki and Cikara, 2015; Strauss et al., 2016; Klimecki, 2019; Kim et al., 2020).

Singer and Klimecki (2014) suggest that an empathic response to suffering can result in two kinds of reactions: *empathic distress*, which is also referred to as personal distress, and *compassion*, which they also referred to as empathic concern. Empathy refers to our general capacity to detect and resonate with others’ emotional states irrespective of their valence. Empathic distress refers to a strong aversive and self-oriented response to others’ suffering, accompanied by the desire to withdraw from an unpleasant situation to protect oneself from excessive negative feelings. On the other hand, compassion is conceived as an openness and connection to a feeling of concern for another person’s suffering – also referred to as *sympathy* – accompanied by the motivation to help (Liotti and Gilbert, 2011). Consequently, it is associated with approach and prosocial motivations (Feldman and Kuyken, 2011; Singer and Klimecki, 2014; Gilbert et al., 2017). For our purpose, it is notable that the field of psychology has seen a surge in compassion research with theoretical, clinical, and educational facets (Strauss et al., 2016; Gilbert, 2017, 2020; Seppelä et al., 2017).

In clinical psychology, the ability to experience oneself as worthy of compassion, and mastering the interpersonal skills that enable one to act compassionately towards others, have become corner stones in novel therapeutic models, such as Compassion Focused Therapy (Gilbert, 2014, 2020). Grounded in evolutionary functional analysis, Gilbert (2019) has conceptualised compassion as sensitivity to suffering in self and others, with a commitment to try to alleviate and prevent it. In his simplified model of emotional regulation, he gives the compassionate mind a central role in dealing with both the restless achievement-focused part and the threat focused and safety-seeking part of the human mind (Gilbert, 2014). According to Gilbert (2014), compassion can generate affiliative experiences, which in turn generate courage to face and engage with difficult, even feared, emotions – including fear of compassion, which has been shown to be related to shame, self-criticism, and depression (Kirby et al., 2019). The academic milieu, in which the supervisor-doctoral student relationship is embedded, is well known for being competitive and hierarchical (Biron et al., 2008; Levecque et al., 2017). Thus, it is very likely that compassion could prevent the eroding effects of this context, perhaps by a jointly creating a *safe base* for the supervisor-student dyad to work from. Furthermore, in an interpersonal space characterised by trust and safety, supervisors and students may be less likely to act unethically and stay true to their values (Dutton et al., 2007; Ozawa-de Silva et al., 2012). Indeed, Gilbert (2014) argues that competencies that generate compassion towards self and others helps us function at our optimum.

The aim of this paper was thus to conceptualise the role of compassion in the supervisor-doctoral student relationship. In the remaining parts of this text, the word *student* will be used with a tacit limitation to a discussion of doctoral students.

## Materials and Methods

To conceptualise the role of compassion in the supervisor-student relationship, we performed a stepwise inquiry of firstly; a search of the literature, secondly; an adaptation of a conceptual model of the supervisor-student relationship, thirdly; an expanded definition and description of the concept of compassion, and fourthly; an analysis of the application of compassion to the conceptual model of the supervisor-doctoral relationship. Lastly, implications for theoretical elaborations and future empirical investigations are discussed.

### Literature Search

The first step of our inquiry into the role of compassion in the supervisor-doctoral student relationship was to perform a literature search. It was performed with systematic use of the following terms in the scientific databases Pubmed, PsychInfo, ScienceDirect, and Google scholar; “compassion and supervision” (+/− “doctoral student” and “PhD student”) “compassion and supervisor and interpersonal relationship”. This search resulted in no empirical articles on the subject. However, we found four references that commented on the topic, which was included as background and context for the analysis.

### The Supervisor-Student Relationship: A Conceptual Model

There is evidence that supervisors’ and doctoral students’ interpersonal relationships have implications for the progress of the work and the satisfaction of the student (Moxham et al., 2013). In the literature of higher education research, there are several models that explicitly or implicitly take interpersonal aspects into account. For example, Gatfield (2005) investigated supervisor “styles” using the two dimensions of structure and support, and Lindén (1999) focused on more narrow factors of freedom vs. control.

**Figure 1:**
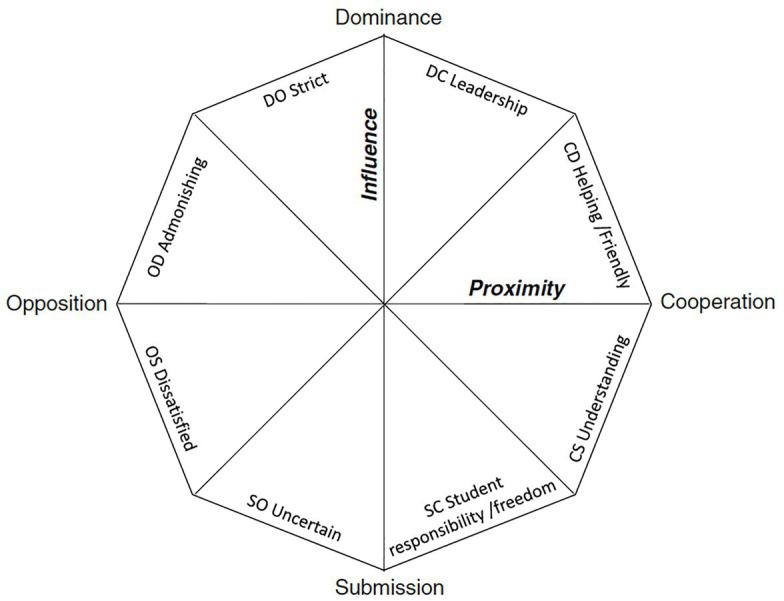
Mainhard et al. (2009) model for supervisor interpersonal behaviour.

For our purpose here, where the challenges and complexities of the relationship are of primary interest, it could be useful to consider the model of Mainhard et al. (2009), which uses a framework from teacher behaviours and evaluates degrees of behaviours on the two independent axes of influence and proximity. The two axes contain eight types of behaviours; leadership, helpfulness/friendliness, understanding, freedom/responsibility, uncertain, dissatisfied, admonishing, and strict (see Figure 1 below). The theoretical underpinnings of this model highlight that the supervisor-student relationship is characterised by bi-directional influences, and that the eight behavioural dimensions are dynamically interdependent. Some behaviours might provoke opposite behaviours, while others provoke similar, creating positive feedback loops. Thus, we view Maynard’s model as a suitable vehicle for a further inquiry into how compassion and compassionate behaviours fit into this framework.

### Compassion: An Operational Definition

One attempt to define compassion and operationalise it in the form of a practical intervention comes from Jazaieri et al. (2014). Compassion, according to the authors, is a multidimensional construct with four facets; (1) An awareness of suffering (in self or others) – a cognitive facet, (2) Sympathetic concern; being emotionally moved by suffering – an affective facet, (3) A wish to see the relief of the suffering – an intentional facet, and (4) A responsiveness or readiness to help relieve that suffering – a motivational facet. Other authors have presented a different order of the facets of compassion. Ozawa-de Silva et al. (2012), for example, placed the affective facet before the subsequent split of cognitive- and attentional facets. Initial studies of a compassion intervention developed by Jazaieri et al. (2014) have lent support to the hypothesis that training in compassion can influence both cognitive and emotional factors that support psychological flexibility and adaptive functioning.

The model from Jazaieri et al. (2014) represents the phenomenon of compassion in an individual, and it requires some extension to be fully applicable to the bi-directional interpersonal relationship between supervisor and student. Gilbert (2014) has argued that prosocial behaviours are sometimes neglected as an important goal for therapeutic change. In addition to overt expressions of compassion, the model also needs a facet that represent what the interaction does to the relationship and what is happening in the interpersonal space shared by supervisor and student. Thus, we suggest that the following two facets should be added for a complete model: (5) Displays of compassion, e.g., facial expressions and prosocial behaviours – a behavioural facet, and (6) A shared sense of warmth and trust – an experiential and relational facet Table 1.

### Compassion and the Supervisor-Student Relationship

The literature search resulted in no empirical references, and only four articles with comments on the subject were deemed of interest for our analysis and discussion. One intriguing finding was the work of Manathunga (2005), mentioned in the introduction. The author presents a program for teaching supervisors called “compassionate rigour”, that seeks to go beyond the administrative framing of supervision into the cognitive and pedagogical domain. However, Manathunga (2005) uses the term *compassion* as a synonym for support, encouragement, and empathy, and does not develop any theoretical framework for the implications of introducing compassion in this context.

A second result from the literature search was a recently published anthology called “The Pedagogy of Compassion at the Heart of Higher Education” (Gibbs, 2017). The book covers a wide variety of topics related to compassion and the challenges of contemporary academic life, including the cultivation of compassion in the classroom (Koutselini, 2017) and faculty leadership (Bresciani-Ludvik, 2017). However, none of the chapters address the topic of student-supervisor-relationship.

A fourth finding was an article termed “Postgraduate supervision: For better or worse”, in which an experienced supervisor in the field of educational research critically elaborates about his changes in behaviours after a systematic and critical self-reflection (Olivier, 2007). The author identifies his directive values as *compassion* and *commitment*, although doubts his ability to live out these stated values. Furthermore, he is not convinced that his students subscribe to the same values and principles. Based on group-discussions with students and written reflections, Olivier (2007) found out that during meetings, both individual sessions, and group meetings, a positive relationship was forged. Based on these unsurprising observations and findings from the literature, Olivier (2007, p. 1138), provides a list of recommendations to fellow supervisors. His fourth advice; “A relationship characterised by mutual love, respect, and obligation should be developed to propagate a less ‘top-down approach’”, validates our interest for the role of compassion in the supervision process. However, neither his observation nor his recommendation elucidates how the two processes are related and mutually influenced by each other. Overall, this limited outcome from the literature search indicates a knowledge gap regarding the role of compassion in the supervisor-doctoral student relationship and that empiric studies are very few or non-existent to date.

### The Potential of Compassion in the Supervisor-Student Relationship

**Figure 2:**
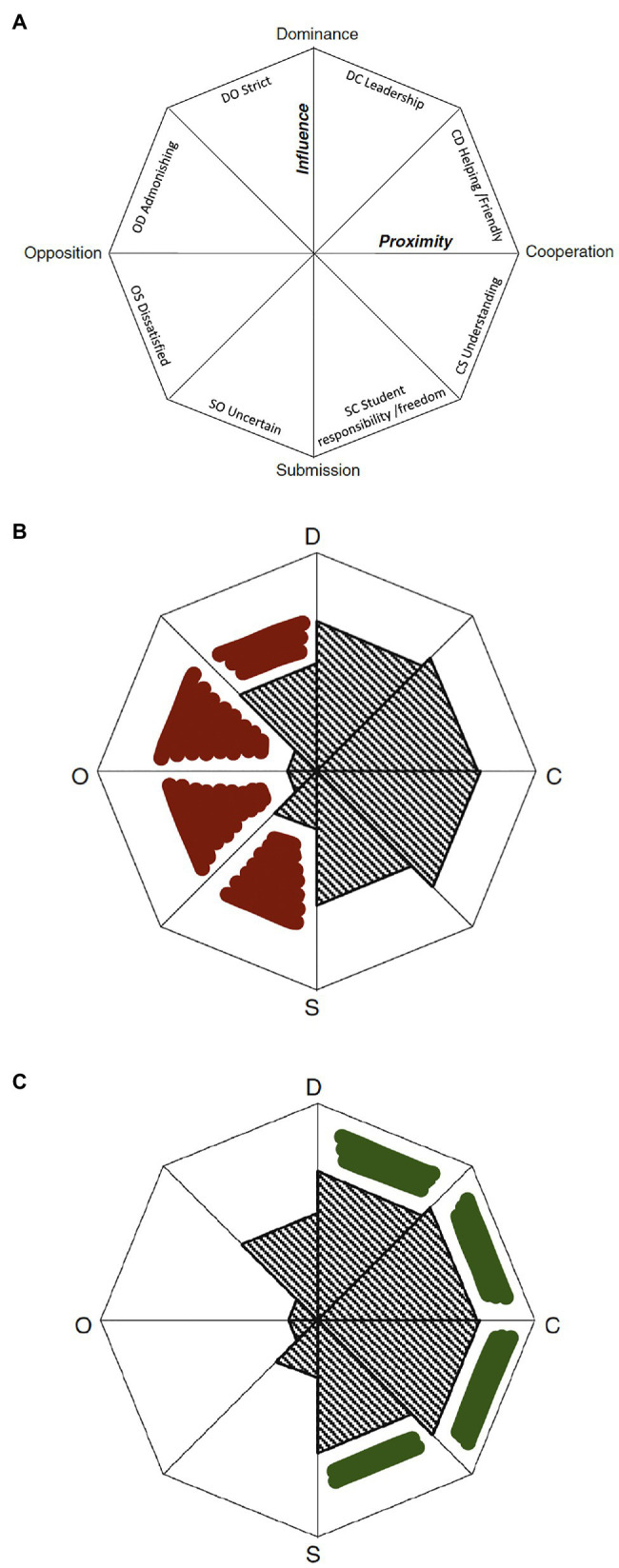
**(A)** Mainhards model of supervisor behaviour. **(B**,**C)** Profiles of average supervisor behaviours in an earlier study (grey) and behaviours hypothetically linked to a lack of compassion (red) and behaviours that theoretically could be enhanced by compassion (green). Adapted from Mainhard et al. (2009).

Here, we proceed with an analysis of the potential role of compassion in the supervisor-student relationship through a theoretical comparison of Mainhard’s model with the extended Jazaieri (2018) definition of compassion. It could be of value to note that a supervisor with low capacity for compassion could not only fail to respond adaptively, he or she could also react with behaviours that are deleterious to the interpersonal relationship and increase the risk of ethical issues and even transgressions (Löfström and Pyhältö, 2014). Examples of such behaviours could be strict or admonishing behaviours, opposition or withdrawal of support, and an increase in submissive behaviours. Figure 2 shows the original Mainhard model and areas of unhelpful and helpful supervisor behaviours that might be influenced by compassion.

In addition to the theoretical basis of compassion as a pro-social phenomenon, that is generally considered a catalyst for closeness, cooperation, and well-being, our analysis includes recent evidence of compassion training’s psychological effects. Interestingly, a review of this literature, which in many aspects is in its infancy, reveal that most studies to date are focused on outcome in the individual and very few report findings in the relational domains (Kirby, 2017; Condon, 2019; Condon et al., 2019; Klimecki, 2019). Considering the pro-social nature of compassion, it is somewhat surprising that the interpersonal domain seems to be understudied. For our purpose here, meaningful outcomes on the individual level represent the starting point, and informed reasoning is then used to bridge the knowledge gap of how these changes translate into the interpersonal space. The following positive and negative outcomes, summarised by Kirby (2017) are influenced by compassion interventions:

- Self-criticism and shame.
- Mind-wandering to unpleasant topics.
- Depressive symptoms and anxiety.
+ Readiness for health behaviour change.
+ Well-being and life satisfaction.
+ Ability to recognise emotions in others.

**Table 1:** Analysis: the application of compassion onto a model for supervisor interpersonal behaviour.

Individual dimensions
(1) **Awareness of suffering** (cognitive)
→Increased ability to recognise emotions and catch early warning signs of stress in self and student foster *understanding* (CS).
→More sensitive to the consequences of harsh or blunt negative feedback can prevent *admonishing* behaviours (OD).
(2) **Sympathetic concern – being emotionally moved by suffering** (affective)
→More accurate empathetic assessment of student’s needs and challenges could calibrate the inner compass for *leadership* style (DC).
→Modelling common humanity and being human could foster bi-directional *understanding* (CS).
→Precursor to signalling a caring attitude could facilitate *trust* (see also 6 below) and *friendliness* (CD).
(3) **A wish to see the relief of the suffering** (intentional) and (4) **A responsiveness or readiness to help relieve that suffering** (motivational)
→Motivating supportive and *helping* measures and directive *leadership* behaviours (CD, DC).
→Possibly a higher threshold for irritation over students’ shortcomings, increased forgiveness, and less *dissatisfied* (DS).
→Prevention of maladaptive reactions against student distress could prevent a drift towards *uncertain* and *dissatisfied* behaviours (OS, SO).
Interpersonal dimensions
(5) **Displays of compassion and overt compassionate behaviours** (behavioural)
→Signalling a caring attitude could facilitate *trust*, which could catalyse processes related to creativity and productivity (CD).
→Create an atmosphere of security that could foster student creativity, productivity, and *responsibility* (SC).
→Inspiring (by model) students to develop *self-compassion*, which could foster *self-leadership* and *responsibility* (DC, SC).
(6) **A shared sense of warmth and trust** (experiential/relational)
→Less depressive symptoms, anxiety, and mind wandering would increase supervisor and student stress resilience, improve *friendliness* and *understanding* (CD, CS), and possibly also decrease self/other *strict criticism* and *dissatisfied* behaviours (DO, OS).
The hypothesised cumulative positive changes in supervisor behaviours are visualised in Figure 2.

## Discussion

The objective of this paper was to conceptualise the phenomenon of compassion in the sometimes strained supervisor-doctoral student relationship. Overall, the literature review revealed that empirical data is largely missing regarding this specific context. We proceeded with our inquiry by analysing possible ways in which compassion could be used to improve supervision, using a theoretical framework based on teacher behaviour (Mainhard et al., 2009). The resulting conceptual framework provides theoretical support to the assumption that compassion may have significant positive impact on the supervisor-doctoral student relationship, both through increases in adaptive supervisor behaviours and decreases in maladaptive behaviours. Although Maynard’s model does not include behaviours that are explicit examples of ethical issues in the relationship, e.g., admonitions, withdrawal of support, and strict behaviours could represent ethical trespasses in specific contexts. The model does also not include outright harmful behaviours such as insulting communication and sexual misconduct. However, since compassion is viewed as a pro-social process, it is plausible to suspect that supervisors with high levels of compassion would be less likely to engage in unethical behaviours (Ozawa-de Silva et al., 2012; Halperin, 2014; Fröding and Osika, 2015; Klimecki et al., 2016; DeSteno et al., 2018). Indeed, based on its central role in many systems of moral thought, and since compassion has been suggested to be an evolved and hardwired moral compass, it has recently been coined “the highest ethics” by researchers in the field of contemplative psychology (Kirby et al., 2017). Perhaps one of the major advantages of a focus on compassion in the supervisor role could be its potential to offer a guiding principle that could radically simplify an immensely complex responsibility.

The literature review revealed that the interpersonal space and relational experiences seem to represent understudied domains of outcome in compassion research. Recently, Gu et al. (2020) developed a tool for assessing compassion, and their definition of the construct overlaps with the extended Jazaieri et al. (2014) model used in our analysis. However, the sixth *interpersonal dimension* (a shared sense of warmth and trust) is not included in the definition or measurement of Gu et al. (2020), and perhaps this dimension needs its own measurement if we are to deepen our empirical understanding. When Gilbert (2014, 2019, 2020) developed Compassion Focused Therapy, he extended the definition of compassion beyond the individual level and described it as an integrated *flow* of compassion. In addition to compassion for others, this model also included becoming receptive to compassion, and compassion towards oneself. However, this model of the tri-directional flow of compassion neglects the interpersonal space domain, in which shared experiences of warmth, trust, and belonging are the fruits of an unimpeded flow of compassion.

**Figure 3:**
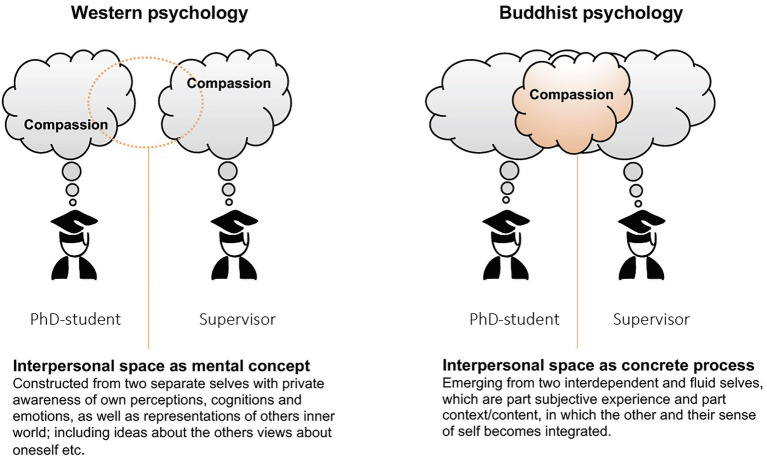
Western- and Buddhist views on the psychology of interpersonal space. Future measurements of e.g., a shared sense of warmth, as fruits of compassionate interactions, will have to address differing views of self and mind.

Further research in this largely uncharted terrain may force scientist to confront philosophical and empirical questions stemming from different views of mind, consciousness, and by extension what constitutes and influence the shared interpersonal space (Varela et al., 2016; Jacobs, 2017; Andriopoulou and Prowse, 2020; Condon and Makransky, 2020). Taking a calculated risk of simplifying complex processes, we propose a model that visualises some crucial differences between western- and Buddhist views on the interpersonal space, which could stimulate future research and reasoning. While western psychology views the individual selves as building blocks in relationship models, a view grounded in Buddhist philosophy would consider the interpersonal space as a process that emerges from two interdependent and fluid selves, that co-arise partly from the interaction (Varela et al., 2016; Jacobs, 2017; Figure 3). Indeed, these differences could have extensive implications for how to conceptualise and measure interpersonal processes and the implications of more or less compassion in the shared space of an ongoing relationship.

One such conceptual challenge is the differences between Western dualism of separate processes of self- vs. other-focused compassion and Buddhist non-dual views, which have implications for both intervention design and choices of assessments in studies with interpersonal processes as outcomes (Quaglia et al., 2020). These philosophical issues might also provide challenges in the empirical investigation of sub-facets of social perception, appraisal, motivation, and action (e.g., emotion regulation), which relate to compassion (Ozawa-de Silva et al., 2012).

Distinctions between compassion and empathy are crucial to have in mind, but there are also lessons to be drawn from empathy research. Despite the limits of empathy (Västfjäll et al., 2014; Bloom, 2017), decades of empirical work have shown a positive association between empathy and pro-social behaviour. In preparation for future studies, researchers should be aware of some of the risks and pitfalls that are often discussed in regard to empathy. One such caveat is that surplus empathy can lead to significant cognitive bias in situations with complex moral dimensions (Västfjäll et al., 2014; Bloom, 2017; Cameron et al., 2019). Increased empathy could also lead to an increased risk of burnout as it might sensitise the individual to suffering without providing the ability to handle the emotional contagion (Singer and Klimecki, 2014). A second common critique is that too much closeness in this specific work relationship could become a burden that distracts from the central academic work (Hockey, 1995).

If researchers of higher education pedagogy would conduct intervention trials, perhaps by sending pairs of supervisors and students to compassion training seminars or retreats, they should consider measuring positive change and also register adverse events (Lindahl et al., 2017; Rozenthal et al., 2018). Future research should also investigate which contextual factors, at the team- (including potential interpersonal challenges with co-supervisors) and management levels of an organisation, could represent obstacles for the growth of compassion in the student-supervisor relationship (Rupprecht et al., 2019). This perspective is highly relevant since the embrace of contemplative practices, such as mindfulness and compassion training programmes, has been criticised for focusing on individual psychological factors, while neglecting contextual factors such as organisational structure and culture (Purser et al., 2016). Indeed, one can assume that organisational factors in the hierarchical, strict, and sometimes penalising academic environment, including harsh competition for limited resources, could hamper supervisor’s capacity for compassion (Biron et al., 2008; Levecque et al., 2017). However, it also seems necessary to study individual differences and predisposing factors for the ability to generate a flow of compassion in the supervisor-student relationship, such as various blocks to compassion (Kirby et al., 2019) and early developed attachment patterns (Mikulincer and Shaver, 2005). Future research should also benefit from a sound scepticism of the current hype surrounding compassion interventions and other contemplative initiatives, and this could be attained by comparing interventions to other methods for cultivating resilience and prosocial functioning (Van Dam et al., 2017).

There are also instances where maladaptive motives and behaviour might simulate genuine compassion that should be studied in this hierarchal context. Catarino et al. (2014) described an important distinction between submissive compassion and genuine compassion, where the submissive kind can be explained as behaving in a helpful way, but where the intention solely is to prevent disruption of the connection to the other person or to be liked, and not from a compassionate motivation. Submissive compassion was associated with shame, depression, anxiety, and stress in the study by Catarino et al. (2014).

This conceptual article certainly raises more questions than it answers, which it is supposed to do. Among the most intriguing questions, that remain to be investigated, is whether supervisor’s and student’s compassion could be influenced through training and how beneficial changes in the relational domain translate into an improvement in student well-being and performance. Indeed, questions about how and what to measure as outcomes from compassion training in this context are of great importance. Perhaps both supervisors and students experience of engagement and sense of meaning should be included.

Interestingly, Whitelock et al. (2008) have shown that a positive interpersonal climate was experienced as one of the chief factors to foster creativity among students and supervisors. Perhaps, in the downstream currents of increased compassion, supervisors and students could find themselves becoming more inventive and original in their thinking and writing.

## Author Contributions

OL conceived the presented idea, including theoretical background regarding supervisor behaviour and performed the systematic literature search. OL and WO provided input on the role of compassion in supervisor-doctorate student relationship, and contributed to the analysis of the results and writing of the manuscript. All authors contributed to the article and approved the submitted version.

### Conflict of Interest

The authors declare that the research was conducted in the absence of any commercial or financial relationships that could be construed as a potential conflict of interest.
